# Total Synthesis and Structure-Activity Relationship of Glycoglycerolipids from Marine Organisms

**DOI:** 10.3390/md12063634

**Published:** 2014-06-18

**Authors:** Jun Zhang, Chunxia Li, Guangli Yu, Huashi Guan

**Affiliations:** Key Laboratory of Marine Drugs, Ministry of Education, School of Medicine and Pharmacy, Ocean University of China, Qingdao 266003, China; E-Mails: junerjasmine@163.com (J.Z.); glyu@ouc.edu.cn (G.Y.); hsguan@ouc.edu.cn (H.G.)

**Keywords:** glycoglycerolipids, marine-origin, bioactivities, total synthesis, structure-activity relationship

## Abstract

Glycoglycerolipids occur widely in natural products, especially in the marine species. Glycoglycerolipids have been shown to possess a variety of bioactivities. This paper will review the different methodologies and strategies for the synthesis of biological glycoglycerolipids and their analogs for bioactivity assay. In addition, the bioactivities and structure-activity relationship of the glycoglycerolipids are also briefly outlined.

## 1. Introduction

**Figure 1:**
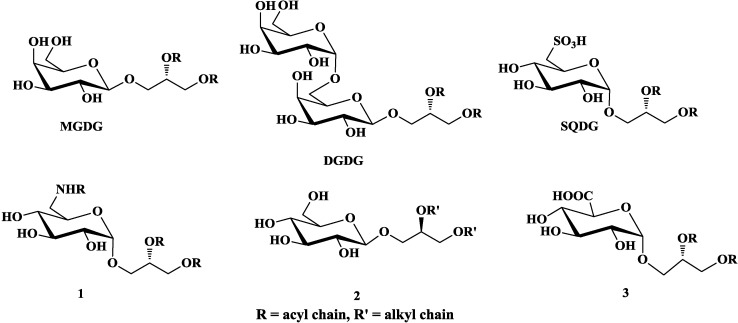
Basic structures of glycoglycerolipids.

Glycoglycerolipids occur widely in marine algae [1,2,3,4], cyanobacteria [5,6,7], and higher plants. The basic structure of glycoglycerolipids is characterized by a 1,2-diacyl-*sn*-glycerol moiety with mono- or oligosaccharide attached at the *sn*-3 position of the glycerol backbone. 1,2-diacyl-3-*O*-(β-d-galactopyranosyl)-*sn*-glycerol (monogalactosyldiacylglycerol, MGDG), 1,2-diacyl-3-*O*-(α-d-galactopyranosyl-(1′→6)-*O*-β-d-galactopyranosyl)-*sn*-glycerol (digalactosyldiacylglycerol, DGDG) and 1,2-diacyl-3-*O*-(6-deoxy-6-sulfo-α-d-glucopyranosyl)-*sn*-glycerol (sulfoquinovosyldiacylglycerol, SQDG) (Figure 1) are common structures in marine cyanobacteria and chloroplasts [8]. A variety of glycoglycerolipids, for example, the sugars other than galactose and glucose (e.g., mannose and rhamnose), occur in α- or β-anomeric configuration and are bound in (1→2), (1→3), (1→4), or (1→6) linkage, are also found in different bacteria [9]. In addition, there are some unique types of glycoglycerolipids including aminoglycoglycerolipids (**1**, Figure 1) [10,11,12,13], ether-linked glycoglycerolipids (**2**, Figure 1) [14,15,16,17,18], and glucuronosylglycerolipids (**3**, Figure 1) [19,20,21] identified from natural products. These natural glycoglycerolipids often possess unusual and sometimes unexpected biological activities, such as anti-tumor [22,23], anti-viral [24,25], and anti-inflammatory activities [26], which make them valuable molecular targets for further investigation. However, the low natural abundance of glycoglycerolipids coupled with the difficulty of isolation hamper their evaluation of bioactivities. Therefore, different synthetic methods are developed to obtain enough pure glycoglycerolipids and analogs for structure-activity relationship study. In the present review, we summarize various methodologies and strategies for the total synthesis of marine glycoglycerolipids. In addition, we describe the important bioactivities of the marine glycoglycerolipids. In addition, some glycoglycerolipids with interesting activities and structures, isolated from other organisms, are also simply introduced to outline an integrated profile of glycoglycerolipids. Finally, the further relationship between the structure and activity is also outlined.

## 2. Bioactivities

### 2.1. Anti-Tumor Activity

**Figure 2:**
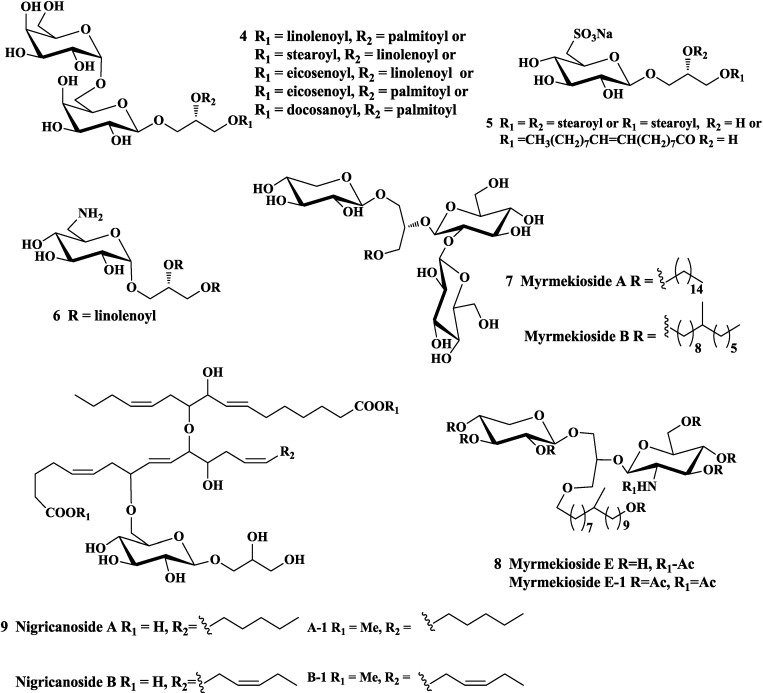
Natural glycoglycerolipids with antitumor activity.

Glycoglycerolipids were screened to have significant anti-tumor activities towards different targets. Sakakibara and co-workers revealed that MGDGs and DGDGs isolated from a cyanobacterium (*Phormidium tenue*) and a freshwater green algae (*Chlorella vulgaris*), showed an inhibitory effect on tumor promoting stage of Epstein-Barr virus-associated early antigen (EBV-EA) activation on Raji cells induced by 12-*O*-tetradecanoylphorbol-13-acetate (TPA) [22,27]. The glycoglycerolipid 1,2-di-*O*-linolenoyl-3-*O*-β-galactopyranosyl-*sn*-glycerol identified from *crassocephalum rabens*, was found to have tumor-suppressive effects on inhibiting TPA-induced expression of COX-2 and nitration of proteins in mouse skin [28]. In 2012, Zhang and co-workers first reported the fatty acid synthase (FAS) inhibition by glycoglycerolipids (**4**, Figure 2) from *O. japonicas*, which provided a scientific basis for using the medicinal plant to treat cancer [29]. In an *in vitro* study and parenteral treatment *in vivo*, the spinach glycoglycerolipid fraction (MGDGs, DGDGs, and SQDGs) potently affected the cancer cells, angiogenesis and solid tumor growth via their inhibition of replicative DNA polymerase activities [30,31,32]. On this basis, Maeda *et al.* [33] further described MGDGs in the spinach glycoglycerolipid fraction could selectively inhibit mammalian replicative polymerase activity, human cultured cancer cell growth, *ex vivo* angiogenesis and *in vivo* solid tumor proliferation.

Recently, naturally occurring sulfoquinovosylglycerolipids have come to be considered promising compounds for cancer therapy and prevention due to their good bioactivities [34]. For example, SQDGs isolated from a marine red algae (*Gigartina tenella*) [35,36], higher plants of pteridophyte (*Athqrium niprmicum*) [37], and spinach [23,38], were proved to have inhibition on replicative DNA polymerase and antiproliferative activity toward human cancer cells. Aoki *et al*. reported that the sulfoquinovosylacylglycerols with C18 fatty acid on the glycerol moiety (**5**, Figure 2) not only inhibited the molecular targets of DNA polymerases but also had the inhibitory effect on the mammalian mitotic centromere-associated kinesin (MCAK) [39]. Moreover, Sahara and co-workers reported that the growth of human adenocarcinoma tumors could be inhibited by 3′-sulfoquinovosyl-1′-monoacylglycerol (SQMG) isolated from sea urchin intestine [40,41]. Their further study demonstrated that the antitumor effect of SQMG could be attributed to antiangiogenic effects, possibly via the downregulation of Tie2 gene expression in SQMG-sensitive tumors [42].

In 2001, the aminoglycoglycerolipid (**6**, Figure 2) isolated from a traditional Chinese medicine, rhizomes of *Serratula strangulate* by Dai and his co-workers, was first reported to exhibit the antitumor activity on mouse melanotic carcinoma cells (B16) [11]. Then, another two aminoglycoglycerolipids extracted from a marine algae were reported to have potent anti-tumor activity towards the strong inhibitory effects on Myt1 kinase (IC_50_ of 0.12 and 0.43 μg/mL), which acted on cell cycle as an important regulator of cdc2/cyclin B kinase activity [13].

In addition, ether-linked glycoglycerolipids isolated from the marine sponge of *Myrmekioderma* sp. were reported to show potent antitumor activity [14,15,16,43,44]. For example, Myrmekiosides A and B (**7**, Figure 2) were able to reverse the phenotype of melanoma H-ras transformed NIH3T3 cells at 5 μg/mL concentration [16]. Peracetylated Myrmekioside E-1 (**8**, Figure 2) derived from ether-linked Myrmekiosides E, showed a significant activity on NSCLC-N6 (IC_50_ *ca.* 7.3 mm) and lung tumor A549 cells (IC_50_ *ca.* 7.3 mm) [15]. Andersen and co-workers obtained a new class of ether-linked glycoglycerolipids, nigricanosides A and B and their respective dimethyl ester derivatives (**9**, Figure 2) from a marine-derived green algae, *Avrainvillea nigricans* [17]. The dimethyl ester A-1 was shown to arrest MCF-7 human breast cancer cells in mitosis with a remarkable IC_50_ of 3 nm and stimulated the polymerization of pure tubulin *in vitro*, and it also inhibited the proliferation of both MCF-7 and human colon cancer HCT-116 cells (IC_50_ of *ca.* 3 nm).

### 2.2. Antiviral Activity

**Figure 3:**
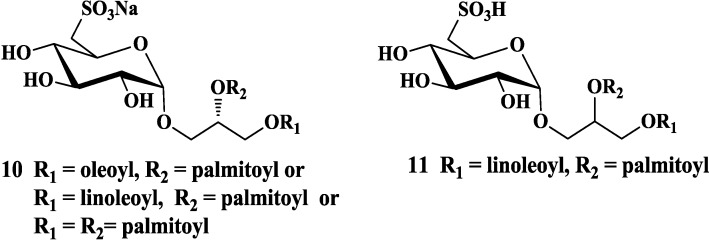
Marine glycoglycerolipids with antiviral activity.

Early in 1989, a series of sulfonic acid-containing glycoglycerolipids isolated from cultured cyanobacteria (blue-green algae), were discovered as a new class of compounds to inhibit the cytopathic effects of the human immunodeficiency virus (HIV-1) [45]. Reshef *et al*. [25,46] reported the capability of similar known and novel diacylated sulfoglycolipids (**10**, Figure 3) to inhibit the DNA polymerase function associated with HIV-1 reverse transcriptase (RT). In recent years, sulfoquinovosylglycerolipids extracted from marine organisms were also reported to be effective on human herpes simplex virus (HSV) infection [47]. For example, sulfoquinovosyl diacylglycerol (**11**, Figure 3) from *Spirulina platensis* exhibited a remarkable activity against HSV-1 with an IC_50_ value of 6.8 μg/mL [24]. Barreto-Bergter and co-workers found that the glycolipids mixture from red algae *Osmundaria obtusiloba* showed anti-viral activity against acyclovir-sensible and acyclovir-resistant HSV-1, and their further study indicated that pure sulfoquinovosyldiacylglycerol exhibited potent antiviral activity against HSV-1 and HSV-2 [48,49].

### 2.3. Anti-Inflammatory Activity

**Figure 4:**
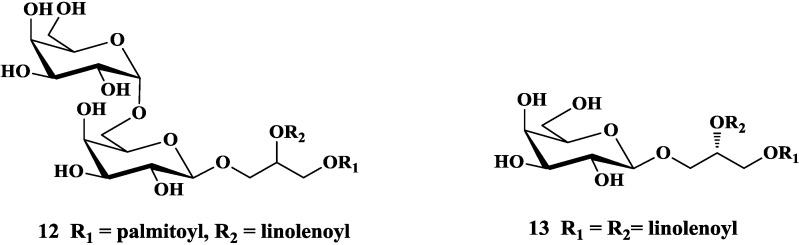
Natural glycoglycerolipids with anti-inflammatory activity.

A crude sulfoquinovosylacylglycerol fraction rich in long-chain polyunsaturated fatty acids (PUFAs) which isolated from the red microalgae *Porphyridium cruentum*, exhibited antioxidant and anti-inflammatory effects because of its ability to inhibit the generation of superoxide anion [26]. In 2005, the purified MGDG, DGDG, and SQDG from cyanobacterium ETS-05 exhibited significant anti-inflammatory activities in two *in vivo* models of inflammation, croton-oil-induced mouse ear oedema and carrageenan-induced mouse footpad oedema [50]. In addition, many glycoglycerolipids isolated from terrestrial plants were reported to have anti-inflammatory activities. For example, a digalactosyl-diacylglycerol (**12**, Figure 4) extracted from *Inula viscosa* was proved to be effective against TPA-induced ear edema in mice and showed notable effect on a multiple-dose murine chronic dermatitis model [51]. Christensen and co-workers discovered galactoglycerolipid (**13**, Figure 4) isolated from the fruit of *Rosa canina* was an anti-inflammatory agent with inhibitory effect on chemotaxis of human peripheral blood neutrophils *in vitro* [52]. Glycoglycerolipids from *Euphorbia nicaeensis* showed significant anti-inflammatory activity on reducing the croton-oil-induced oedema response [53].

### 2.4. Other Activities

**Figure 5:**
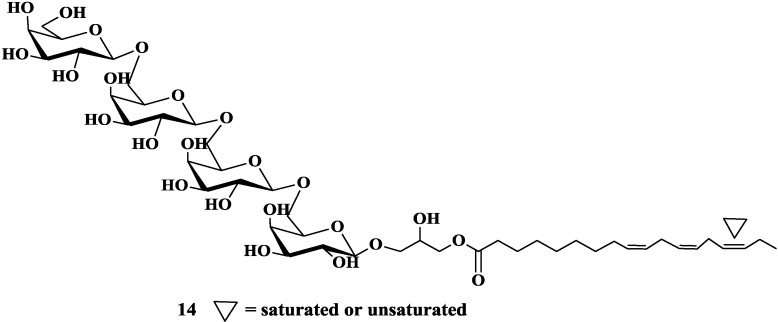
Natural glycoglycerolipids with other activities.

In addition to the bioactivities mentioned above, glycoglycerolipids from various sources were found to have some other activities. For example, the bacterial monogalactosyldiacylglycerol M874B, 1,2-di-*O*-(12-methyltetradecanoyl)-3-*O*-β-d-galactopyranosyl-*sn*-glycerol, was characterized as an alkyl peroxyl radical scavenger and also capable of protecting cells from death caused by heating and exogenous H_2_O_2_ [54,55]. Two new tetrasaccharide glycoglycerolipids (**14**, Figure 5) obtained from pumpkin (*Cucurbita moschata*) demonstrated significant glucose-lowering effect in streptozotocin- and high-fat-diet-induced diabetic mice [56]. Moreover, the fraction of the olive oil rich in glycoglycerolipids was detected to have high levels of platelet activating factor (PAF) antagonist to present protective effect against atherosclerosis [57].

## 3. Total Synthesis

As noted above, glycoglycerolipids isolated from eukaryotic algae, cyanobacteria and even higher plants showed wide spectrum of biological and pharmacological activities. However, only limited quantities of pure glycoglycerolipids can be obtained from natural sources and the similar structures of glycoglycerolipids with same polarity and size make them difficult to separate as a single compound [58]. Hence, synthetic glycoglycerolipids are necessary for biological evaluation. The main challenge in the synthesis of glycoconjugate is to control the stereoselectivity in the glycosylation reaction. For glycoglycerolipids, both the anomeric configuration of the glycosidic linkage and the chirality of the glycerol moiety need to be considered in the synthesis process. In the following section, various total synthetic strategies, which utilize different glycosyl donors, such as glycosyl halide, trichloroacetimidate, thioglycoside, and so on, to react with the appropriate glycerol derivatives in the glycosylation, are summarized for the synthesis of different types of glycoglycerolipids.

### 3.1. Total Synthesis of Mono- and Di-Glycosyldiacylglycerols

**Scheme 1:**
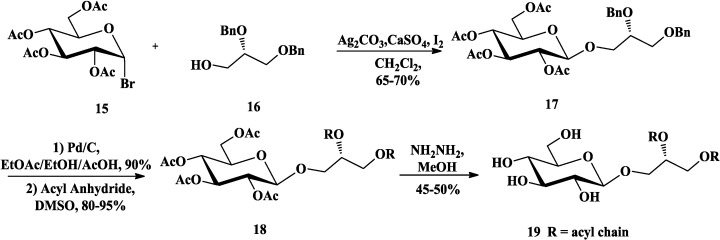
Mannock synthesis of monoglycosyldiacylglycerol with β-glycoside.

**Scheme 2:**
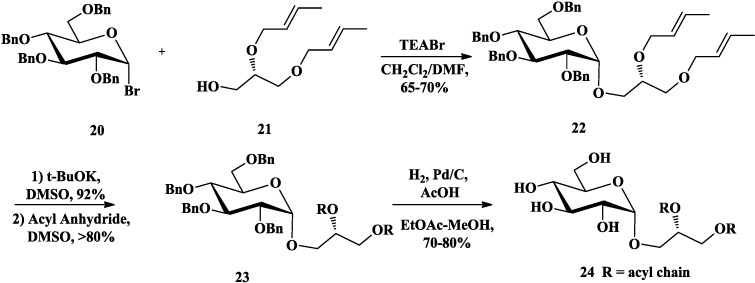
Mannock synthesis of monoglycosyldiacylglycerol with α-glycoside.

Mono- and di-glycosyldiacylglycerols are major membrane lipids in blue green algae and higher plants [59]. The structural architecture involves a glycosidic linkage between the sugar and C-3 of glycerol and ester linkage between fatty acids and two hydroxyls of glycerol, where galactose is the common sugar (MGDG, DGDG, Figure 1). Mannock and co-workers had synthesized a homologous series of glycoglycerols with odd- and even-numbered hydrocarbon chains ranging in length from 10 to 20 carbon atoms to investigate their physical properties [60,61,62]. The synthesis of 1,2-diacyl-3-*O*-β-d-glucopyranosyl-*sn*-glycerols and corresponding α-glycoside are reported respectively as examples to introduce their work (Scheme 1 and Scheme 2). Generally, the Koenigs-Knorr method using acyl glycosyl halide as glycosyl donor and silver oxide/iodine as the catalyst was adopted to construct β-d-glycoside in synthesis of the glycoglycerolipids [58,63,64]. However, intramolecular migration usually occurred when 1,2-*O*-isopropylidene-*sn*-glycerol was used as the acceptor under Koenigs-Knorr method [65]. Thus, Mannock *et al*. [61] chose the appropriately blocked alcohol **16** as the acceptor, which was stable to the reaction conditions and easily prepared from d-mannitol (Scheme 1). The glycosylation followed by the hydrogenolysis and acylation of **17** under mild conditions gave **18**. Treatment of **18** with hydrazine hydrate could selectively remove the acetate groups in the presence of the fatty acyl groups to afford monoglycosyldiacylglycerol **19** [66]. On the other hand, α-d-glycoside in the glycoglycerolipids could be constructed using halide-ion catalyzed condensation of 2,3,4,6-tetra-*O*-benzyl-d-glucosyl bromide (**20**, Scheme 2) with 1,2-*di*-*O*-(but-2-enyl)-*sn*-glycerol (**21**). Then, the base-catalyzed elimination of the but-2-enyl groups and acylation afforded **23**, followed by the catalytic hydrogenation, to give the final products **24**.

**Scheme 3:**
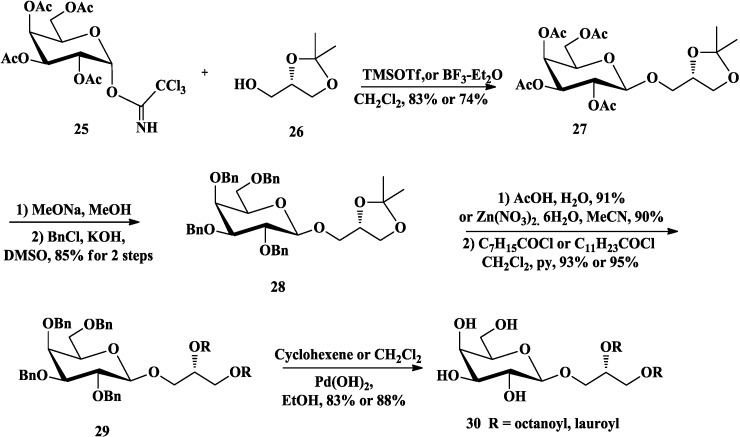
Lafont synthesis of MGDG.

**Scheme 4:**
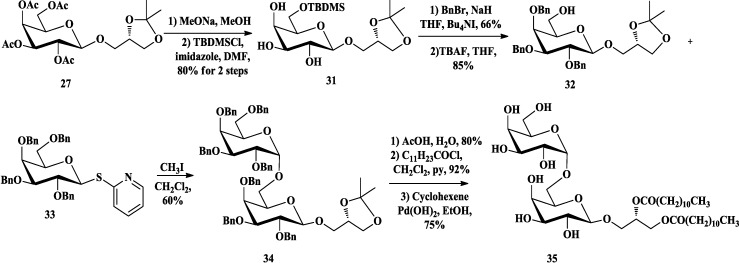
Lafont synthesis of DGDG.

Compared with galactosyl bromides [67], the trichloroacetimidate methodology of glycosylation developed by Schmidt [68], which allowed reaction with chiral 1,2-*O*-isopropylidene-*sn*-glycerol without racemization, was also common in the synthesis of glycoglycerolipids [66,69,70]. Lafont *et al*. [71,72,73] had synthesized MGDGs (**30**, Scheme 3) and the related DGDG (**35**, Scheme 4) based on this trichloroacetimidate methodology to examine the substrate specificity of a purified pancreatic lipase-related protein 2 (PLRP2). They prepared 2,3,4,6-tetra-*O*-acetyl-α-d-galactopyranosyl trichloroacetimidate (**25**) [74] as the donor to accomplish the glycosylation with (*S*)-1,2-*O*-isopropylidene-*sn*-glycerol (**26**) in the presence of trimethylsilyl trifluoromethanesulfonate (TMSOTf) [72] or boron trifluoride etherate [71]. After the acetyl protecting groups were converted to benzyl (Bn) groups, the isopropylidene group of **28** was removed and the fatty esters were then introduced by an excess of acyl chloride. Catalytic hydrogenation of the Bn groups gave the final compounds **30**. The related DGDG **35** [73] was prepared from compound **27** [72], which was then deacetylated and selectively silylated at 6-OH using tert-butyldimethylsilyl chloride (TBDMSCl). The resulting **31** was benzylated and the silyl ether was cleaved with tetra-*n*-butylammonium fluoride (TBAF) to provide the alcohol **32**. Glycosylation of acceptor **32** with **33** [75] in the promoter of methyl iodide was able to induce the α-anomeric stereoselectivity. After acidolysis, esterfication and deprotection, final DGDG (**35**) was successfully obtained.

**Scheme 5:**
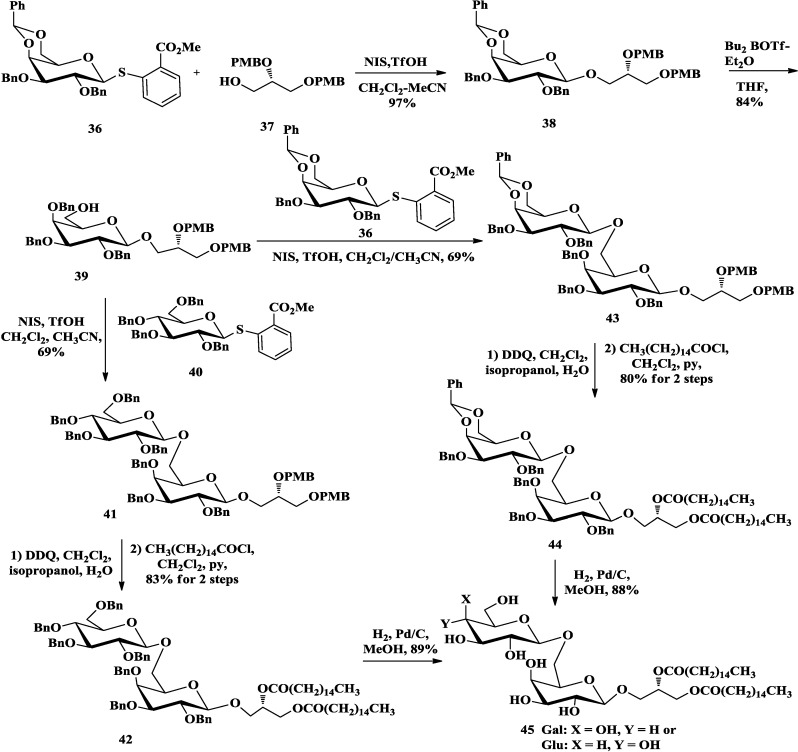
Nishida synthesis of diglycosyldiacylglycerol.

In 2009, β-Gal(1→6)Gal and β-Glu(1→6)Gal diglycosyldiacylglycerols (**45**, Scheme 5) from *M. pneumoniae* were synthesized by Nishida and co-workers [76]. They employed a non-malodorous thioglycosylation methodology [77] to promote β-glycosylation reactions. 2-methoxycarbonylphenyl 1-thioglycosides protected with *O*-benzyl groups (**36**, **40**), and chiral *sn*-glycerol **37** which was used as the acceptor to avoid racemization, were chosen for the synthesis of diglycosyldiacylglycerols. All the glycosylations were processed with iodosuccinimide (NIS) and trifluoromethanesulfonic acid (TfOH) in a mixture of acetonitrile and dichloromethane (1:1) to give desired β-selectivity. After two times of glycosylation, the β-isomer of the disaccharides (**41**, **43**) were easily separated. After PMB groups on the glycerol moiety were cleaved with DDQ, palmitoyl groups were introduction by palmitoyl chloride to furnish **42** and **44**. Finally, catalytic hydrogenation afforded the diglycosyldiacylglycerols **45**.

**Scheme 6:**
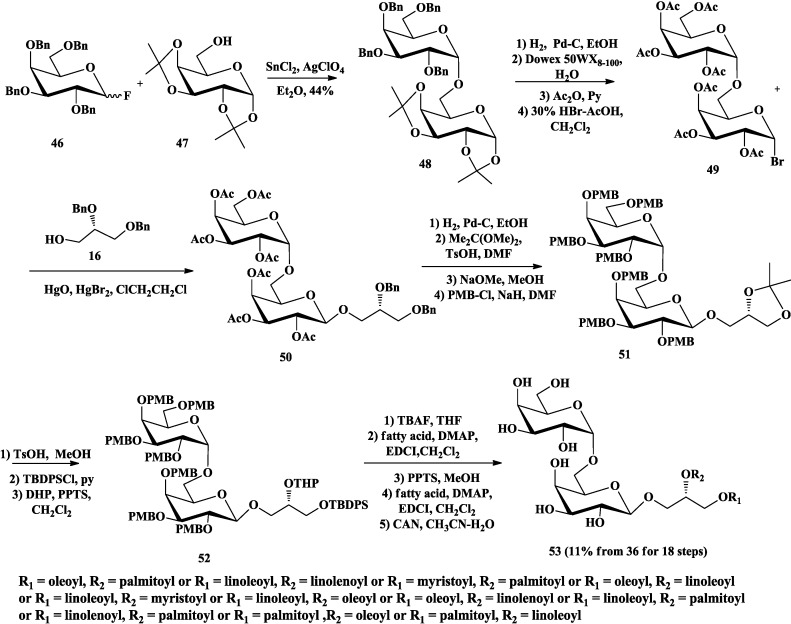
Goda synthesis of DGDG.

Compared with Lafont and Nishida’s methods of synthesizing DGDG, Goda and co-workers reported another strategy, which prepared the α-Gal(1→6)Gal disaccharide **48** first, then coupled with the glycerol moiety to synthesize DGDG **53** (Scheme 6) [78]. After all protecting groups of disaccharide **48** were converted to acetyl groups, **49** was reacted with **16** in the presence of HgO and HgBr_2_ to give **51** as β-anomer. Then, Bn groups on the glycerol moiety of **51** were converted to isopropylidene group and the acetyl groups at the sugar moiety were converted to *p*-methoxybenzyl (PMB) ethers. The hydroxyl groups at glycerol moiety were protected individually with *t*-butyldiphenylsilyl (TBDPS) and tetrahydropyranyl (THP) ether (**52**) after the removal of the isopropylidene group. Different acyl groups were incorporated at *sn*-1 and *sn*-2 position respectively after TBDPS and THP ether were removed. Finally, the PMB protecting groups were removed by treatment with ceric ammonium nitrate (CAN) to afford DGDGs (**53**) with different acyl groups.

### 3.2. Total Synthesis of Sulfoquinovosylacylglycerols

**Scheme 7:**
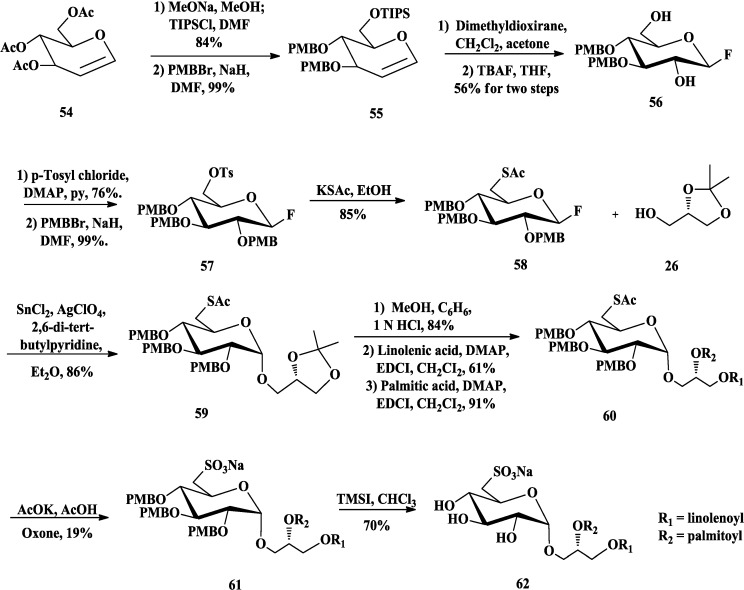
Danishefsky synthesis of SQDG.

The sulfoquinovosylacylglycerols (SQAGs) are one of the abundant sulfur-containing glycerolipids in the natural world. SQAGs are characterized by their unique sulfonic acid head group, a 6-deoxy-6-sulfo-glucose, referred as sulfoquinovose [79,80]. Due to the notable anti-HIV activities of sulfolipids isolated from cyanobacterial (blue-green algae) media [45], Danishefsky and Gordon developed a synthetic route to obtain the related cyanobacterial sulfolipids [65]. Starting from tri-*O*-acetyl-d-glucal **54** (Scheme 7), deacetylation and protection with TIPS (tri-isopropylsilyl) and PMB afforded derivative **55**. Oxidation of **55** followed by the reaction with TBAF afforded anomeric β-fluoro-glucosyl **56**. The primary alcohol of **56** could be distinguished by tosylation with TsCl and the hydroxyl at C-2 was converted to its 2-*O*-PMB derivative **57**. Then, the Ts function suffered displacement with treatment of potassium thioacetate (KSAc) to give **58** [81]. The anomeric fluoride **58** was coupled with glycerol derivative **26** in the presence of stannous chloride (SnCl_2_), silver (I) perchlorate (AgClO_4_) and 2,6-di-tert-butylpyridine, to generate **59** without racemization. After the isopropylidene group was cleaved, esterfication of the glycerol hydroxyls with different fatty acids afforded **60**. Finally, a sulfonic acid was introduced at C-6 by oxidation of **60** with Oxone followed by the removal of PMB groups to give **62** as sodium salt.

**Scheme 8:**
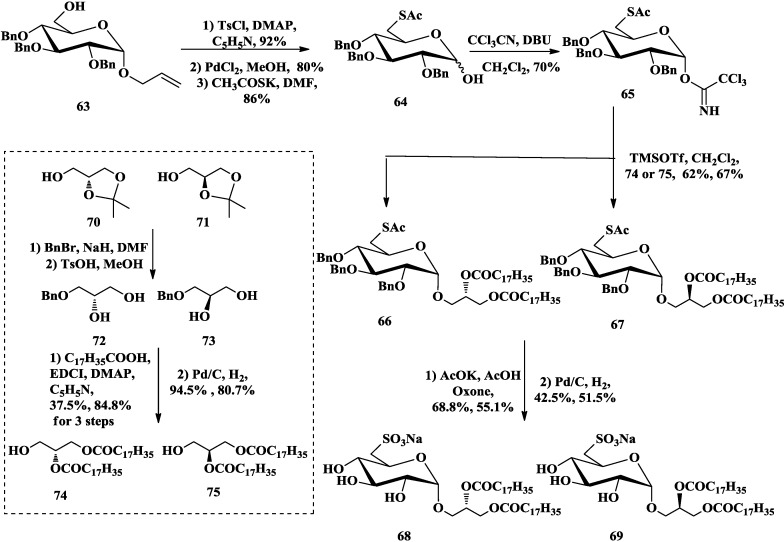
Hanishima synthesis of SQDGs with different chirality.

Applying the same method to introduce sulfonic acid to C-6 of the sugar, Hanishima *et al*. reported chiral synthetic strategy as follows to obtain SQDGs with different stereochemistry at *sn*-2 of the glycerol moiety (Scheme 8) [82]. Tosylation and deallylation of the starting allyl glycoside **63** [83], and subsequent nucleophilic displacement of the tosyloxy group generated **64**. Then α-trichloroacetimidate **65** was obtained as the glycosyl donor, and fatty acid esters of the chiral glycerols were employed as the glycosyl acceptors (**74**, **75**) to avoid racemization. Glycosylation reactions processed in the presence of TMSOTf to give the desired α-glycoside **66** and **67**. Finally, oxidation of the resulting compounds and removal of the protecting groups afforded chiral SQDGs **68** and **69**, respectively.

**Scheme 9:**
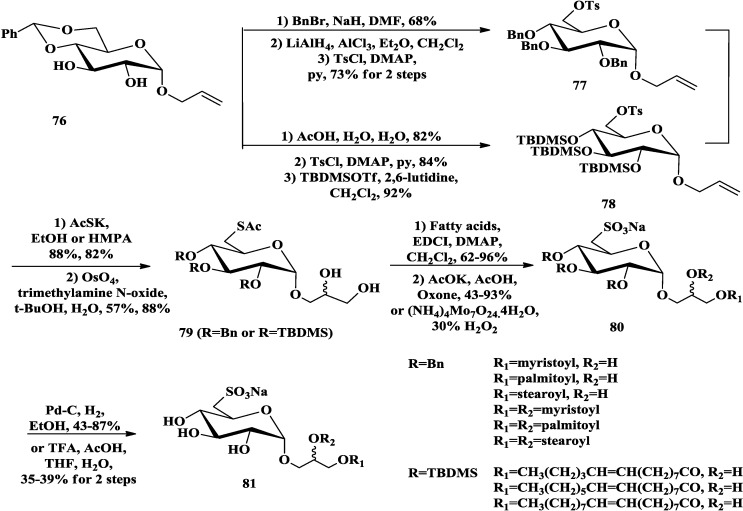
Hanishima synthesis of SQDGs and SQMGs.

Hanishima *et al*. [84] also synthesized diastereomeric SQDGs and SQMGs (sulfoquinovosylmonoacylglycerols) bearing diverse fatty acids. They protected the secondary hydroxyl groups of the allyl glucopyranoside **76** [85] (Scheme 9) with Bn and TBDMS respectively, then, the tosyloxyl group converted into thioacetyl group to achieve sulfolipids with saturated and unsaturated fatty acid. Different from the strategy mentioned above, allyl group at the anomeric site was α-selectively introduced to construct C-3 back glycerol moiety by oxidation with OsO_4_. Acylation of the diastereomeric diol **79** with saturated or unsaturated fatty acids gave the corresponding mixture of mono- and diesters, which could be easily separated on silica gel column. The resulting compounds were oxidated to **80**, followed by deprotection with Pd-C or trifluoroacetic acid (TFA) to afford the final SQDGs and SQMGs (**81**).

### 3.3. Total Synthesis of Aminoglycoglycerolipids

**Scheme 10:**
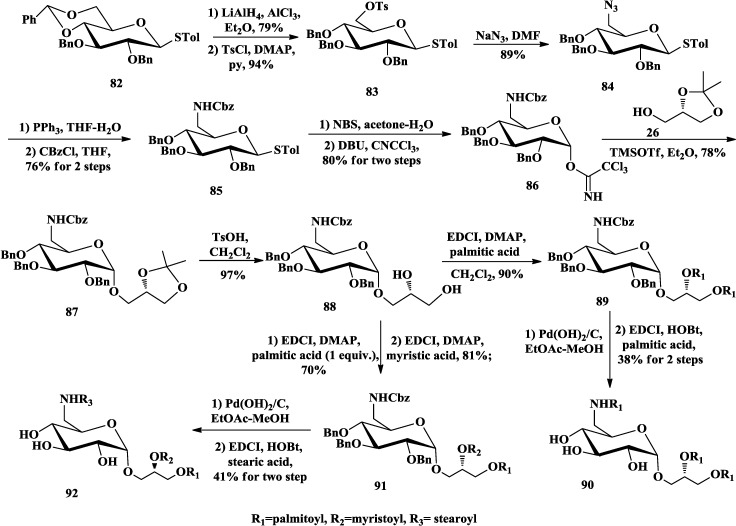
Li synthesis of aminoglycoglycerolipids.

The aminoglycoglycerolipids (**1**, Figure 1) attracted much attention because of their unique bioactivities [10,11,12,86,87,88]. Their structures involve an α-d-6-deoxy-6-aminoglucoside linked to C-3 of glycerol. In 2008, Li and co-workers reported a facile method for the synthesis of the two natural aminoglycoglycerolipids **90** and **92** (Scheme 10), which was reported to inhibit human Myt1-kinase [13,89]. Selective removal of the benzylidene of protected thioglycoside **82** and tosylation of the primary hydroxyl at C-6 gave **83**, which was converted to its azide derivative **84** by nucleophilic reaction with sodium azide. Afterwards the azide group was reduced to amino group and protected with benzylcarbonyl (Cbz) group to give **85**. The key step was selective α-glycosylation of trichloroacetimidate donor **86** with **26** in diethyl ether (Et_2_O). Removal of acetonide group of **87** and acylation with different ratios of diverse fatty acids gave **89** and **91**. Then deprotection of Bn and Cbz groups and selective acylation of the amino group afforded **90** and **92**.

**Scheme 11:**
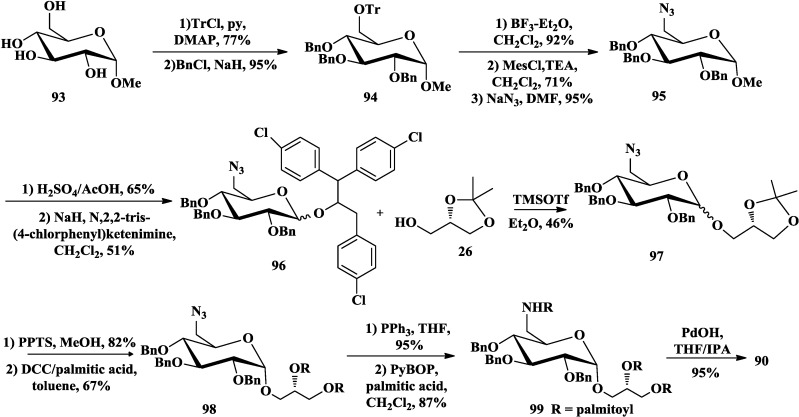
Schmidt synthesis of aminoglycoglycerolipid.

Schmidt and co-workers also described the total synthesis of aminoglycoglycerolipid **90** [13,90]. They employed triphenylmethyl (Tr) to selectively protect the primary hydroxyl group of α-methylglucopyranoside **93** (Scheme 11) and the remaining free hydroxyl groups were protected with Bn. Afterwards, the trityl protecting group was converted into azide **95**, which was not only a function group to provide amino, but also a stable protecting group. To achieve good stereoselectivity of the glycosylation, **95** was converted to glucosyl donor **96** and reacted with **26** in the catalyst of TMSOTf to form **97** in an anomer mixture (α:β = 78:22). A better separation of the anomers can be achieved by hydrolysis of the raw isopropylidene-protecting group. Then, the glycerol hydroxyl groups of the α-anomer were acylated with palmitic acid by dicyclohexylcarbodiimide (DCC) method. Reduction of the azido group of **98** using the Staudinger reaction followed by acylation of the amino group afforded **99**. Final product **90** was obtained by removal of the protecting groups.

**Scheme 12:**
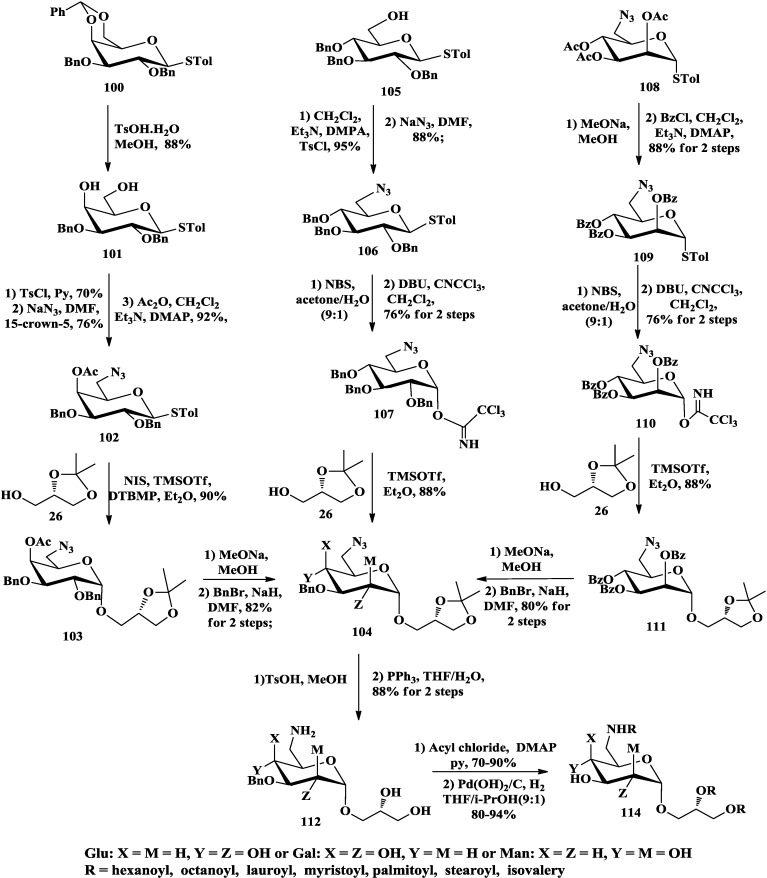
Li synthesis of aminoglycoglycerolipids with diverse glycosyls and fatty acids.

In addition, Li and co-workers completed the synthesis of aminoglycoglycerolipids with different glycosyl and fatty acids for biological activity assay [91,92,93]. The azide functional group at C-6 of the glycosyl was introduced by nucleophilic reaction with sodium azide (Scheme 12). Constructing α-glycoside of 6-deoxy-6-aminoglycoglycerolipids by glycosylation reactions with different glycosyl (d-gluco-, d-galacto-, d-manno-) moiety were not consistent. Among them, glucosyl trichloroacetimidate donor **107** was used to prepare α-anomeric glucoside at low substrate concentration in Et_2_O (α:β = 33:1). The 4-*O*-acetyl protecting group of galactosyl donor **102** was employed as remote neighboring participation group to give the galactoside with high α-anomeric selectivity (α:β = 32:1) in the glycosylation. While utilizing neighboring group participation, mannosyl trichloroacetimidate donor **110** with 2-*O*-benzoyl protecting group was applied to obtain absolute α-anomeric mannosyl glycoside. Afterwards, hydrolysis of the isopropylidene and reduction of the azido afforded the key intermediates **112**. Acylation of the amino and hydroxyl groups processed with different acyl chloride in pyridine followed by deprotection of Bn to give a series 6-deoxy-6-aminoglycoglycerolipids **114**.

**Scheme 13:**
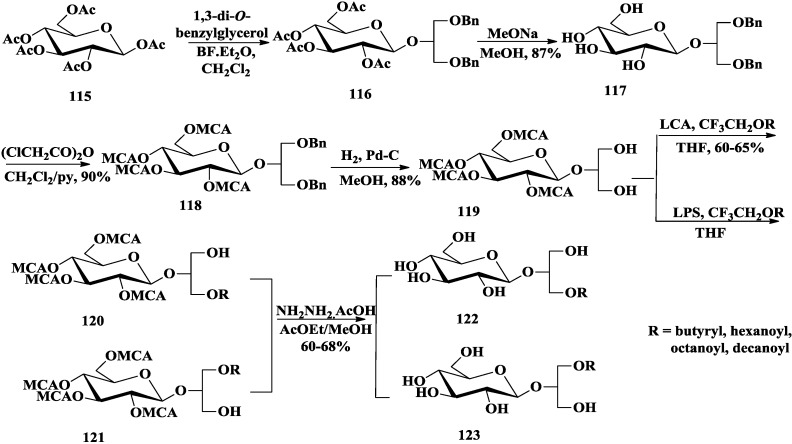
Colombo chemoenzymatic synthesis of (2*S*)/(2*R*)-l-*O*-acylglycoglycerolipids.

**Scheme 14:**
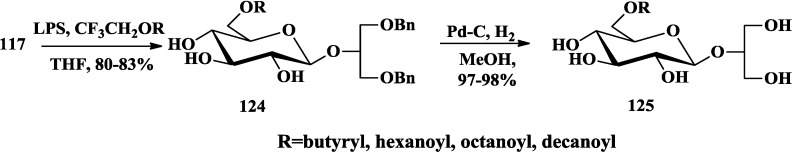
Colombo chemoenzymatic synthesis of 6′-*O*-acylglycoglycerolipids.

**Scheme 15:**
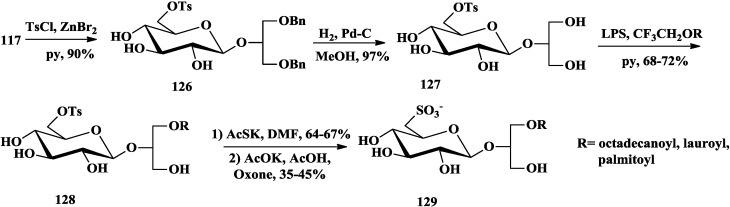
Colombo chemoenzymatic synthesis of sulfoquinovosylglycerolipids.

**Scheme 16:**
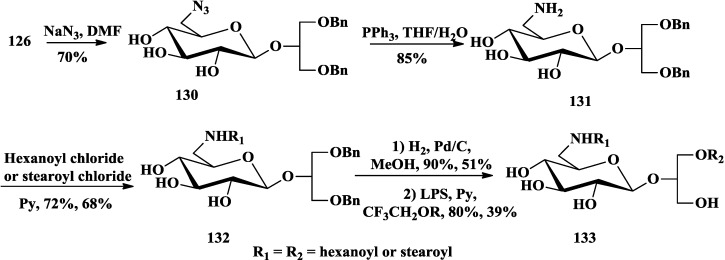
Colombo chemoenzymatic synthesis of 6-amino-6-deoxyglycoglycerolipids.

Although natural glycoglycerolipids, in which the sugar is linked to the *sn-*3 of the glycerol, have attracted attention as anti-tumor-promoting compounds [13,22,27,42]. Colombo and co-workers proved that synthesized 2-*O*-β-d-glycopyranosyl-*sn*-glycerol had bioactivities comparable with the corresponding 1-*O*- and 3-*O*-isomers [94]. They synthesized different 2-*O*-β-d-glycoglycerolipid derivatives by chemoenzymatic method and tested their antitumor promoting effect [95,96,97,98]. Different from the chemical acylation methods mentioned above, all of the glycoclycerolipids including monoacylglycoglycerolipids **122** and **123** [98,99] (Scheme 13), 6′-*O*-acylglycoglycerolipids **125** [98] (Scheme 14), sulfoquinovosylglycerolipids **129** [95] (Scheme 15) and 6-amino-6-deoxy-glycoglycerolipids **133** [97] (Scheme 16), were easily monoacylated using enzymes as catalysts and 2,2,2-trifluoroethyl esters of various long chain carboxylic acids as acyl carriers (CF_3_CH_2_OR). Two enzymes, *Pseudomonas cepacia* (LPS) and *Candida antarctica* (LCA) lipases, were proved highly regio- and diastereoselectivity, while the former yielded the 2*S*-l-*O*-acetyl derivative and the latter yielded its (2*R*)-diastereoisomer [99,100].

## 4. The Structure-Activity Relationship

Generally, the important biological activities are related to their chemical feature. Many studies showed that the bioactivities of the glycoglycerolipids are associated with the structures including the sugar moiety, the position of the glycerol linkage to the sugar, the length and location of the acyl chain, and the anomeric configuration of the sugar.

For example, Colombo and co-workers have done a great job for synthesizing different glycoglycerolipids to evaluate the influence of the structure on the anti-tumor-promoting activity. They tested the antitumor promoting effect on EBV-EA activation induced by the tumor promoter TPA and achieved the preliminary conclusion as follows. First, integral structure of the glycoglycerolipid was essential for the bioactivity. Replacing the 6-oxygen of the sugar moiety by a nitrogen or sulfur atom [95,97,101], and substitution of the glycerol with a methyl group could greatly reduce the inhibition [94]. Second, the length of the acyl chains which linked to the hydroxyl of the glycerol moiety through ester bonds was more important for the activity, rather than the position of the ester function and the nature of the sugar [102]. The inhibition effect could reach the maximum when the glycoglycerol connected with hexanoyl (C6) chain [103,104]. Moreover, the type of the acyl chain also played an important role on anti-tumor-promoting activity, where the branched acyl chains linked to the glycerol enhanced the activity, and the presence of an aliphatic or aromatic ring exerted a negative effect [105]. In addition, the site of attachment of the sugar to the glycerol moiety, the anomeric configuration and introduction of another acyl chain onto hydroxyl at C-6′ of the sugar or not, seemed to play negligible roles on improving the antitumor potential [94,98,104,106].

Based on the inhibitory effect of DNA polymerase α and β of the natural sulfoquinovosylacylglycerol, Sakaguchi, Mizushina, and their co-workers, synthesized many derivatives of SQAG to examine the structure-inhibitory function relationship. They demonstrated that not only the chain length of the fatty acid, but also the sulfate moiety of the glycerolipids was important for the inhibitory activity [84,107,108,109]. In addition, the anomeric configuration was not important for the inhibitory effect [110], and the inhibition effect could be maintained when 6-sulfo-d-quinovosylacylglycerol was replaced by a 6-sulfo-d-fucosylacylglycerol or 6-sulfo-d-rhamnosylacylglycerol, which indicated that species of the sugar also had little effect on the inhibition [109]. Interestingly, SQDG had a stronger inhibitory effect on both polymerases *in vitro* compared with SQMG, however, the further investigation on a human cancer cell line (NUGC-3) showed that SQMG showed potent growth inhibitory effect on the cancer cells while none of the SQDG tested had such an effect, suggesting that the SQDG can not penetrate into the cells [109].

In addition, Li and co-workers have synthesized series of aminoglycoglycerolipids to test their inhibitory activity of Myt 1-kinase [91,92]. The preliminary results indicated that the aminogalactoglycerolipid series had better inhibition than the corresponding glucose series, meaning that the sugar moiety of the aminoglycoglycerolipids played an important role on inhibiting Myt 1-kinase. Moreover, although the presence of longer length of fatty acids (C16–C18) caused an enhancing of the inhibitory activity, the branched acyl chains showed the best inhibition against Myt 1-kinase [91].

## 5. Conclusions

Various types of glycoglycerolipids isolated from natural products and their wide range of bioactivities have attracted much attention. However, there are challenges of separating the single product and acquiring different type of glycoglycerolipids for evaluating their activity-structure relationship. This review covered the activities of the naturally occurring glycoglycerolipids, total synthesis and their special relationship of structure-activity. The present synthetic methodologies and strategies provided diverse natural and unnatural glycoglycerolipids, including MGDG/DGDG, SQDG/SQMG and aminoglycoglycerolipids for bioactivity evaluation. The biological results of the glycoglycerolipids acting on different models indicate that most bioactivities are closely related with the sugar moiety and acyl chain of the glycoglycerolipids. More structural modification and further mechanism studies are in progress, which will facilitate a great understanding of glycoglycerolipids as candidate drugs.
